# Ultrasound examiners' ability to describe ovarian cancer spread using preacquired ultrasound videoclips from a selected patient sample with high prevalence of cancer spread

**DOI:** 10.1002/uog.29208

**Published:** 2025-04-18

**Authors:** D. Fischerova, P. Pinto, M. Pesta, M. Blasko, M. C. Moruzzi, A. C. Testa, D. Franchi, V. Chiappa, J. L. Alcázar, M. Wiesnerova, D. Cibula, L. Valentin, collaborators, J. l. Alcázar, G. Bolomini, F. Ciccarone, C. Codecà, I. de Blasis, E. Epstein, A. Farulla, D. Franchi, F. Frühauf, I. Guiggi, V. Chiappa, R. Kocian, M. Leombroni, M. Ludovisi, F. Mascilini, F. Moro, A. P. Pinto, F. Pozzati, L. Quagliozzi, H. Reina, A. C. Testa, L. Valentin, D. Verri, V. Weinberger

**Affiliations:** ^1^ Gynecologic Oncology Centre, Department of Gynecology, Obstetrics and Neonatology, First Faculty of Medicine Charles University and General University Hospital in Prague Prague Czech Republic; ^2^ Department of Gynecology Portuguese Institute of Oncology Francisco Gentil Lisbon Portugal; ^3^ First Faculty of Medicine Charles University and General University Hospital in Prague Prague Czech Republic; ^4^ Faculty of Mathematics and Physics Charles University Prague Czech Republic; ^5^ Department of Computer Science, Faculty of Electrical Engineering Czech Technical University in Prague Prague Czech Republic; ^6^ Gynecologic Oncology Unit, Department of Woman and Child Health and Public Health Fondazione Policlinico Universitario A. Gemelli IRCCS Rome Italy; ^7^ Section of Obstetrics and Gynecology University Department of Life Sciences and Public Health, Università Cattolica del Sacro Cuore Rome Italy; ^8^ Preventive Gynecology Unit, Division of Gynecology European Institute of Oncology IRCCS Milan Italy; ^9^ Department of Gynecologic Oncology Fondazione IRCCS Istituto Nazionale dei Tumori Milan Italy; ^10^ Department of Obstetrics and Gynecology, Clinica Universidad de Navarra University of Navarra Pamplona Spain; ^11^ QuironSalud Hospital Málaga Spain; ^12^ Masaryk University Institute of Biostatistics and Analyses Brno Czech Republic; ^13^ Department of Obstetrics and Gynecology Skåne University Hospital Malmö Sweden; ^14^ Department of Clinical Sciences Malmö Lund University Lund Sweden

**Keywords:** diagnostic imaging, education, gynecology, inter‐rater agreement, ovarian cancer, reliability, staging, training, ultrasound, video recordings

## Abstract

**Objectives:**

To assess the ability, as well as factors affecting the ability, of ultrasound examiners with different levels of ultrasound experience to detect correctly infiltration of ovarian cancer in predefined anatomical locations, and to evaluate the inter‐rater agreement regarding the presence or absence of cancer infiltration, using preacquired ultrasound videoclips obtained in a selected patient sample with a high prevalence of cancer spread.

**Methods:**

This study forms part of the Imaging Study in Advanced ovArian Cancer multicenter observational study (NCT03808792). Ultrasound videoclips showing assessment of infiltration of ovarian cancer were obtained by the principal investigator (an ultrasound expert, who did not participate in rating) at 19 predefined anatomical sites in the abdomen and pelvis, including five sites that, if infiltrated, would indicate tumor non‐resectability. For each site, there were 10 videoclips showing cancer infiltration and 10 showing no cancer infiltration. The reference standard was either findings at surgery with histological confirmation or response to chemotherapy. For statistical analysis, the 19 sites were grouped into four anatomical regions: pelvis, middle abdomen, upper abdomen and lymph nodes. The videoclips were assessed by raters comprising both senior gynecologists (mainly self‐trained expert ultrasound examiners who perform preoperative ultrasound assessment of ovarian cancer spread almost daily) and gynecologists who had undergone a minimum of 6 months' supervised training in the preoperative ultrasound assessment of ovarian cancer spread in a gynecological oncology center. The raters were classified as highly experienced or less experienced based on annual individual caseload and the number of years that they had been performing ultrasound evaluation of ovarian cancer spread. Raters were aware that for each site there would be 10 videoclips with and 10 without cancer infiltration. Each rater independently classified every videoclip as showing or not showing cancer infiltration and rated the image quality (on a scale from 0 to 10) and their diagnostic confidence (on a scale from 0 to 10). A generalized linear mixed model with random effects was used to estimate which factors (including level of experience, image quality, diagnostic confidence and anatomical region) affected the likelihood of a correct classification of cancer infiltration. We assessed the observed percentage of videoclips classified correctly, the expected percentage of videoclips classified correctly based on the generalized linear mixed model and inter‐rater agreement (reliability) in classifying anatomical sites as being infiltrated by cancer.

**Results:**

Twenty‐five raters participated in the study, of whom 13 were highly experienced and 12 were less experienced. The observed percentage of correct classification of cancer infiltration ranged from 70% to 100% depending on rater and anatomical site, and the median percentage of correct classification for the 25 raters ranged from 90% to 100%. The probability of correct classification of all 380 videoclips ranged from 0.956 to 0.975 and was not affected by the rater's level of ultrasound experience. The likelihood of correct classification increased with increased image quality and diagnostic confidence and was affected by anatomical region. It was highest for sites in the pelvis, second highest for those in the middle abdomen, third highest for lymph nodes and lowest for sites in the upper abdomen. The inter‐rater agreement of all 25 raters regarding the presence of cancer infiltration ranged from substantial (Fleiss kappa, 0.68 (95% CI, 0.66–0.71)) to very good (Fleiss kappa, 0.99 (95% CI, 0.97–1.00)) depending on the anatomical site. It was lowest for sites in the upper abdomen (Fleiss kappa, 0.68 (95% CI, 0.66–0.71) to 0.97 (95% CI, 0.94–0.99)) and highest for sites in the pelvis (Fleiss kappa, 0.94 (95% CI, 0.92–0.97) to 0.99 (95% CI, 0.97–1.00)).

**Conclusions:**

Ultrasound examiners with different levels of ultrasound experience can classify correctly predefined anatomical sites as being infiltrated or not infiltrated by ovarian cancer based on video recordings obtained by an experienced ultrasound examiner, and the inter‐rater agreement is substantial. The likelihood of correct classification as well as the inter‐rater agreement is highest for sites in the pelvis and lowest for sites in the upper abdomen. However, owing to the study design, our results regarding diagnostic accuracy and inter‐rater agreement are likely to be overoptimistic. © 2025 The Author(s). *Ultrasound in Obstetrics & Gynecology* published by John Wiley & Sons Ltd on behalf of International Society of Ultrasound in Obstetrics and Gynecology.

## INTRODUCTION

According to European consensus, contrast‐enhanced computed tomography (CE‐CT), whole‐body diffusion‐weighted magnetic resonance imaging (WB‐DWI/MRI) and positron emission tomography‐CT with a structured radiology report are options for the initial evaluation of patients with advanced tubo‐ovarian carcinoma (herein denoted as ‘ovarian cancer’)^1^. Ultrasound examination by an expert sonographer can also be used to assess ovarian cancer spread^1^. In experienced hands, ultrasound can correctly detect cancer infiltration of the peritoneum and lymph nodes and predict non‐resectability of a tumor^2^, ^3^, ^4^. For ultrasound to be a viable alternative to other imaging modalities, it has long been recommended to perform systematic examination of all peritoneal surfaces, abdominal visceral organs and retroperitoneal and inguinal lymph nodes, ideally using a structured report, and, more recently, examination of the superior diaphragmatic (cardiophrenic) lymph nodes has also been recommended^5^. However, ultrasound quality and efficacy are operator dependent. Ideally, ultrasound examinations to assess the spread of cancer should be performed by trained operators in specialized cancer centers. To our knowledge, there is no published study describing the ability of ultrasound examiners with different levels of ultrasound experience to correctly detect infiltration of ovarian cancer in different anatomical locations, and none describing the inter‐rater agreement regarding cancer infiltration.

The aims of this study were: (1) to estimate the ability of ultrasound examiners with different levels of ultrasound experience to correctly detect infiltration of ovarian cancer in predefined anatomical locations; (2) to identify factors affecting the examiner's ability to correctly detect ovarian cancer infiltration; and (3) to estimate the inter‐rater agreement regarding the presence or absence of ovarian cancer infiltration, using preacquired ultrasound videoclips obtained from a selected patient sample with a high prevalence of cancer spread.

## METHODS

This study reports the results of a secondary aim of the Imaging Study in Advanced ovArian Cancer (ISAAC) multicenter observational study (ClinicalTrials.gov, NCT03808792). The primary aim of the ISAAC study was to compare the performance of abdominopelvic ultrasound with that of CE‐CT and WB‐DWI/MRI in the preoperative prediction of the non‐resectability of ovarian cancer. The study was performed in accordance with current guidelines of the International Council for Harmonisation on Good Clinical Practice (ICH‐GCP; ichgcp.net), the seventh revision of the Declaration of Helsinki and applicable regulatory and country‐specific requirements. Ethical approval for the ISAAC study was obtained from the Ethics Committee of the General University Hospital in Prague, Czech Republic (29/18, 04.06.2018). The Guidelines for Reporting Reliability and Agreement Studies (GRRAS) were followed^6^.

### Ultrasound examination and collection of videoclips

For the purpose of this study, which was conducted between January 2020 and December 2021, the principal investigator of the ISAAC study (D.F.) prospectively collected videoclips of a standardized and structured ultrasound examination for assessing the spread of ovarian cancer in consecutive patients with suspected ovarian cancer enrolled into the ISAAC study at her center. D.F. is an expert ultrasound examiner with more than 20 years' experience of scanning patients with gynecological cancer, including estimation of cancer spread. The ultrasound machine used was a Voluson E10 (GE Healthcare, Zipf, Austria), equipped with a 5–9‐MHz RIC5‐9 transducer, a 2–9‐MHz convex array transducer and a 6–15‐MHz linear array transducer. The ultrasound examination included 19 anatomical sites grouped into four anatomical regions (pelvis, middle abdomen, upper abdomen, lymph nodes) (Table 1). Infiltration of cancer in five of the 19 sites would indicate non‐resectable disease: (1) small intestine surface; (2) small intestine mesentery; (3) lesser omentum; (4) hepatic hilum; and (5) liver parenchyma (central or multisegmental metastasis surrounded by normal parenchyma between liver visceral surface and lesion)^7^. Videoclips ranging from 20 s to 45 s in duration were collected from each of the 19 sites. The goal was to obtain 10 videoclips with and 10 without cancer infiltration for each site. When 10 video recordings showing cancer infiltration and 10 showing no cancer infiltration from a particular site had been obtained, we stopped collecting recordings from this site and continued collecting only from sites with fewer than the required number of videoclips. All videoclips were edited to remove information that could be used directly for patient identification, and a body marker and an indicator of the position of the ultrasound probe were added. The reference standard was used to confirm the presence or absence of disease before the videoclips were uploaded onto an online platform for analysis by different raters. Videoclips from patients with histological diagnoses other than ovarian cancer were excluded.

**Table 1:** Anatomical sites (*n* = 19), grouped into four anatomical regions, which were evaluated by raters for presence of ovarian cancer infiltration in abdomen, pelvis and groin

Anatomical region/specific site
Pelvis
Anterior compartment*
Posterior compartment†
Rectosigmoid wall
Mesorectum and sigmoid mesocolon‡
Middle abdomen
Greater omentum
Anterior abdominal wall
Left and right paracolic gutters
Colon surface
Small intestine surface§
Mesentery of small intestine§,¶
Upper abdomen
Left and right diaphragm
Spleen**
Liver parenchyma§
Liver surface
Hepatic hilum§,††
Lesser omentum (hepatogastric/hepatoduodenal ligament)§
Lymph nodes
Inguinal
Abdominal (para‐aortic)
Pelvic (para‐iliac)

* Anterior compartment includes bladder, vesicouterine pouch and anterior uterine wall.

† Posterior compartment includes uterine fundus, posterior uterine wall, pouch of Douglas, rectosigmoid, mesorectum and sigmoid mesocolon.

‡ Mesorectum and sigmoid mesocolon correspond to space between posterior wall of colon (rectum and sigmoid colon) and sacral bone.

§ Infiltration of small intestine surface or mesentery, liver parenchyma (central or multisegmental metastasis surrounded by normal parenchyma between liver visceral surface and lesion), hepatic hilum or lesser omentum indicates non‐resectable tumor.

¶ Mesenteric deep involvement was due either to lymphatic spread from para‐aortic lymph nodes alongside visceral branches of aorta (i.e. superior and inferior mesenteric vessels) or to peritoneal involvement of deeply infiltrating mesenteric root.

** Splenic involvement was defined as peritoneal carcinomatosis on surface and/or intraparenchymal metastases.

†† Hepatic hilum involvement was due either to lymphatic spread from para‐aortic lymph nodes alongside visceral branches of aorta (i.e. celiac trunk) or to peritoneal involvement.

### Reference standard

The reference standard was intraoperative findings supplemented by histological analysis of surgical specimens, or of core‐needle biopsy samples if primary surgery was not possible (e.g. for liver metastases). If neither biopsy nor surgery was possible, suspected metastases were followed up with imaging (ultrasound or CE‐CT) during specific oncological treatment (chemotherapy, biological treatments or a combination) to assess treatment response. Partial or complete regression in platinum‐sensitive disease, or progression in platinum‐refractory or platinum‐resistant disease, was considered to confirm cancer infiltration and was used as an alternative reference standard.

### Rater selection

Ultrasound examiners (raters) were invited in writing to assess the videoclips regarding cancer infiltration. They were recruited from ovarian cancer surgery centers where systematic ultrasound examination is used routinely before surgery to evaluate the spread of the disease. The invited raters were either senior gynecologists who had gained their ultrasound skills mainly by self‐training and who performed preoperative ultrasound assessment of ovarian cancer spread almost daily, or gynecologists with at least Level‐II ultrasound competence in gynecological scanning plus a minimum of 6 months' supervised training in preoperative ultrasound assessment of ovarian cancer spread in a center for ovarian cancer surgery in which ultrasound is routinely used for this purpose. According to the European Federation for Societies of Ultrasound in Medicine and Biology (EFSUMB), a Level‐II practitioner must have undertaken at least 2000 gynecological ultrasound examinations and must perform at least 500 gynecological ultrasound examinations per year^8^. Raters are listed at the end of this article.

To assess their ultrasound experience, each rater completed a questionnaire regarding their level of expertise in gynecological scanning (defined as EFSUMB Level II or III); number of scans to assess the extent of ovarian cancer that they usually perform annually; number of years during which they had been performing abdominal and pelvic ultrasound examinations to assess ovarian cancer spread; type of training in ultrasound assessment of the extent of ovarian cancer (self‐trained or by trainers in a center specializing in ovarian cancer surgery, and number of months in training); whether they performed abdominopelvic ultrasound examinations routinely to evaluate ovarian cancer spread before surgery; and type of ultrasound center in which they work (oncological referral center *vs* other). Raters with at least 10 years' experience of performing ultrasound examinations for the assessment of ovarian cancer spread (i.e. ovarian cancer staging) or who performed at least 100 scans for ovarian cancer staging annually were classified as highly experienced. Raters with less than 10 years' experience of performing ultrasound examinations for the assessment of ovarian cancer spread and with fewer than 100 scans for ovarian cancer staging performed annually were classified as less experienced.

### Evaluation of videoclips

The videoclips were uploaded onto an online platform created for the study by a bioengineer (M.B.). Before starting to assess the videoclips, the raters attended a 60‐min live‐streamed webinar organized by the principal investigator, which explained the platform and the ultrasound methodology used to assess the extent of ovarian cancer spread. After attending the webinar, the raters received an e‐mail with a link to the platform. After individual log‐in to the platform from their own computer, each rater evaluated each videoclip regarding: (1) cancer infiltration (yes/no); (2) image quality (numeric rating scale ranging from 0 to 10, where 0 = very unsatisfactory and 10 = perfect); and (3) diagnostic confidence (numeric rating scale ranging from 0 to 10, where 0 = very uncertain and 10 = absolutely certain). The raters were blinded to clinical information including patient demographics and any imaging results. However, they were aware that for each anatomical site there would be 10 videoclips showing cancer infiltration and 10 not showing cancer infiltration, since this was specified in the study protocol. They were allowed 3 months to complete individually their evaluations of all videoclips and they were able to rewatch the videoclips and revise their answers during this period. Answers were transferred automatically to the central database using a ‘save’ button. Completeness of the evaluations was checked automatically by the platform software, and study progress was monitored by the bioengineer. After all the evaluations had been completed by an individual rater, they were transferred automatically to the central database for subsequent analysis with no possibility of further change.

### Statistical analysis

Formal sample‐size calculation was performed for the primary endpoint of the ISAAC study but not for this secondary endpoint. We report the observed percentage of videoclips classified correctly. Continuous data are given as mean ± SD, or as median, interquartile range and range.

To estimate the likelihood of correct classification regarding ovarian cancer infiltration and to estimate which factors may affect this likelihood, we used a generalized linear mixed model with random effects. This was used both as a prediction model and as a model to explain which variables affected the likelihood of correct classification. The outcome variable was correct classification of the videoclip (yes or no; Bernoulli variable). The following were tested as variables in the model: diagnostic confidence of the rater; image quality according to the rater; anatomical region (pelvis, middle abdomen, upper abdomen, lymph nodes); the five sites that, if infiltrated, would indicate non‐resectability of the tumor; rater's level of expertise in gynecological scanning (EFSUMB level); annual number of ultrasound examinations performed to assess ovarian cancer spread; number of years performing ultrasound examinations to assess ovarian cancer spread; level of ultrasound experience in ovarian cancer staging (highly *vs* less experienced); type of training in ultrasound assessment of the extent of ovarian cancer (self‐trained, or by trainers in a center specializing in ovarian cancer surgery, including length of training in months); whether routinely using ultrasound to evaluate cancer spread before surgery; and type of ultrasound center in which rater worked (oncological referral center *vs* other). The fixed effects were image quality, level of diagnostic confidence and anatomical region. The random effects were random intercept and level of diagnostic confidence. The intercept in our generalized linear mixed model corresponded to the log odds for the probability of correct classification for a hypothetical rater and hypothetical scan with the baseline characteristics (i.e. image quality = 0, level of confidence = 0 and anatomical region = ‘lymph nodes’). Traditional stepwise model‐building was employed, i.e. from the starting full model, the non‐significant covariates (variables) were removed in a stepwise fashion. Final model pruning was performed in a backward–forward selection by considering the regressors' interactions. A *P*‐value of 0.05 was used to decide whether to keep a variable in the model. From the final generalized linear mixed model, the Bayes' prediction of the probability of correct classification was calculated for each rater using the estimated fixed parameters and taking into account the random effects in the model.

Inter‐rater agreement for classifying videoclips as showing cancer infiltration or not is reported as Fleiss kappa^9^ (with 95% CI) for each of the 19 sites. A kappa value of 0.81–1.00 was taken to indicate very good agreement, 0.61–0.80 substantial agreement, 0.41–0.60 moderate agreement, 0.21–0.40 fair agreement and ≤ 0.20 poor agreement^10^.

Statistical calculations were performed using *R* statistical software by R Core Team (2022) version 4.2.2 (released on 2022‐10‐31) (*R* Foundation, Vienna, Austria).

## RESULTS

During the study period, 134 patients underwent ultrasound imaging for assessment of the spread of suspected ovarian malignancy. Videoclips of 96 of the 134 patients were needed to complete the collection of 190 videoclips showing cancer infiltration in 19 sites and 190 videoclips showing no cancer infiltration in these sites. Twelve (12.5%) of the 96 women had International Federation of Gynecology and Obstetrics^11^ Stage‐I disease, four (4.2%) had Stage‐II disease, 41 (42.7%) had Stage‐III disease and 39 (40.6%) had Stage‐IV disease. Fourteen patients with metastases in the liver parenchyma (*n* = 10) or spleen (*n* = 4), who could not undergo surgical exploration or biopsy, were followed up with CE‐CT during systemic treatment to investigate the reaction of the metastatic disease to treatment.

### Raters

The raters were 25 gynecologists from 14 centers in six countries (Italy, Sweden, Czech Republic, Switzerland, Portugal, Spain). All raters reported that they were Level‐II or Level‐III gynecological ultrasound examiners according to EFSUMB^8^. Using our definitions, 13 raters were highly experienced and 12 were less experienced in performing ultrasound examination to assess ovarian cancer spread (Table S1). Five raters had achieved their ultrasound skill for evaluating ovarian cancer spread by self‐training, and 20 had undergone supervised training in a gynecological oncological center for at least 6 months.

### Performance of raters

The observed percentage of videoclips classified correctly regarding cancer infiltration, raters' diagnostic confidence and raters' assessment of image quality are summarized in Table 2 and Figure S1, and details are given in Tables S2–S6. The observed percentage of videoclips classified correctly ranged from 70% to 100% depending on rater and anatomical site (Tables S2–S5). The median percentage of correctly classified videoclips for the 25 raters, computed from their individual percentages of correctly classified videoclips, was 100% for sites in the pelvis, 98.3% for sites in the middle abdomen, 94.2% for sites in the upper abdomen, 96.7% for lymph‐node metastases and 96.0% for sites that, if infiltrated, would indicate non‐resectability of the tumor (Table S6). Differences between the highly experienced and less experienced raters were minimal. The mean rater diagnostic confidence using the numeric rating scale was 9.3 for sites in the pelvis, 8.8 for sites in the middle abdomen, 8.5 for sites in the upper abdomen, 9.1 for lymph‐node metastases and 8.5 for the five sites that, if infiltrated, would indicate non‐resectability of the tumor, and the mean image quality scores for these anatomical regions and the five non‐resectable sites were, respectively, 8.8, 8.6, 8.3, 8.7 and 8.4 (Table 2).

**Table 2:** Percentage of 380 ultrasound videoclips classified correctly regarding ovarian cancer infiltration in abdomen, pelvis and groin by 25 raters, rater's diagnostic confidence and image quality according to rater, for each of 19 anatomical sites

	All raters (*n* = 25)			Highly experienced raters (*n* = 13)			Less experienced raters (*n* = 12)		
Anatomical site	Videos classified correctly (%)	Diagnostic confidence level (NRS)	Image quality (NRS)	Videos classified correctly (%)	Diagnostic confidence level (NRS)	Image quality (NRS)	Videos classified correctly (%)	Diagnostic confidence level (NRS)	Image quality (NRS)
All 380 videoclips	96.6 ± 5.65 [70–100] 100 (95–100)	8.8 ± 1.52 [0–10]	8.6 ± 1.54 [0–10]	97.1 ± 5.2 [75–100] 100 (95–100)	9.1 ± 1.39 [0–10]	8.7 ± 1.53 [0–10]	95.9 ± 6.04 [70–100] 100 (95–100)	8.5 ± 1.59 [0–10]	8.4 ± 1.53 [1–10]
Pelvis									
Anterior compartment	98.4 ± 3.45 [90–100] 100 (100–100)	9.1 ± 1.36 [3–10]	8.4 ± 1.9 [2–10]	98.8 ± 3.00 [90–100] 100 (100–100)	9.3 ± 1.28 [4–10]	8.6 ± 1.73 [2–10]	97.9 ± 3.96 [90–100] 100 (99–100)	9.0 ± 1.41 [3–10]	8.2 ± 2.04 [2–10]
Posterior compartment	99.8 ± 1.00 [95–100]100 (100–100)	9.2 ± 1.23 [2–10]	8.7 ± 1.49 [3–10]	100 ± 0 [100–100] 100 (100–100)	9.3 ± 1.35 [2–10]	8.8 ± 1.43 [4–10]	99.6 ± 1.44 [95–100] 100 (100–100)	9.2 ± 1.08 [3–10]	8.6 ± 1.54 [3–10]
Rectosigmoid wall	99.4 ± 2.2 [90–100] 100 (100–100)	9.4 ± 0.98 [5–10]	9.1 ± 1.17 [3–10]	99.6 ± 1.39 [95–100] 100 (100–100)	9.6 ± 0.87 [5–10]	9.1 ± 1.23 [3–10]	99.2 ± 2.89 [90–100] 100 (100–100)	9.2 ± 1.04 [5–10]	9 ± 1.09 [4–10]
Mesorectum and sigmoid mesocolon	99.0 ± 2.5 [90–100] 100 (100–100)	9.3 ± 1.1 [5–10]	9.1 ± 1.13 [4–10]	100 ± 0 [100–100] 100 (100–100)	9.5 ± 1.04 [5–10]	9.2 ± 1.08 [5–10]	97.9 ± 3.34 [90–100] 100 (95–100)	9.1 ± 1.13 [5–10]	8.9 ± 1.16 [4–10]
Pelvis, all four sites	99.2 ± 2.47 [90–100] 100 (100–100)	9.3 ± 1.18 [2–10]	8.8 ± 1.48 [2–10]	99.6 ± 1.67 [90–100] 100 (100–100)	9.4 ± 1.16 [2–10]	8.9 ± 1.41 [2–10]	98.6 ± 3.05 [90–100] 100 (100–100)	9.1 ± 1.17 [3–10]	8.7 ± 1.54 [2–10]
Middle abdomen									
Greater omentum	99.0 ± 3.23 [85–100] 100 (100–100)	9.2 ± 1.35 [0–10]	8.9 ± 1.39 [0–10]	99.6 ± 1.39 [95–100] 100 (100–100)	9.3 ± 1.43 [0–10]	9.0 ± 1.42 [0–10]	98.3 ± 4.44 [85–100] 100 (100–100)	9.1 ± 1.25 [2–10]	8.7 ± 1.33 [2–10]
Anterior abdominal wall	98.6 ± 3.07 [90–100] 100 (100–100)	9.2 ± 1.19 [2–10]	9.0 ± 1.23 [2–10]	98.5 ± 3.15 [90–100] 100 (100–100)	9.4 ± 1.1 [5–10]	9.2 ± 1.25 [3–10]	98.8 ± 3.11 [90–100] 100 (100–100)	9 ± 1.25 [2–10]	8.8 ± 1.18 [2–10]
Left and right paracolic gutters	95.8 ± 3.44 [90–100] 95 (95–100)	8.5 ± 1.64 [1–10]	8.3 ± 1.47 [3–10]	96.5 ± 3.76 [90–100] 95 (95–100)	8.9 ± 1.49 [3–10]	8.3 ± 1.62 [3–10]	95 ± 3.02 [90–100] 95 (95–95)	8 ± 1.69 [1–10]	8.3 ± 1.28 [4–10]
Colon surface	97.4 ± 5.42 [75–100] 100 (95–100)	8.5 ± 1.57 [2–10]	8.3 ± 1.38 [5–10]	96.9 ± 6.93 [75–100] 100 (95–100)	8.7 ± 1.61 [2–10]	8.3 ± 1.45 [5–10]	97.9 ± 3.34 [90–100] 100 (95–100)	8.3 ± 1.5 [4–10]	8.2 ± 1.29 [5–10]
Small intestine surface*	97.0 ± 5.00 [80–100] 100 (95–100)	8.7 ± 1.61 [0–10]	8.7 ± 1.40 [2–10]	98.8 ± 2.19 [95–100] 100 (100–100)	9.1 ± 1.36 [5–10]	8.7 ± 1.44 [3–10]	95 ± 6.40 [80–100] 97.5 (90–100)	8.4 ± 1.78 [0–10]	8.6 ± 1.36 [2–10]
Mesentery of small intestine*	97.8 ± 3.56 [90–100] 100 (95–100)	8.7 ± 1.58 [0–10]	8.4 ± 1.45 [2–10]	97.3 ± 4.39 [90–100] 100 (95–100)	9.1 ± 1.21 [4–10]	8.6 ± 1.4 [5–10]	98.3 ± 2.46 [95–100] 100 (95–100)	8.3 ± 1.81 [0–10]	8.3 ± 1.50 [2–10]
Middle abdomen, all six sites	97.6 ± 4.13 [75–100] 100 (95–100)	8.8 ± 1.53 [0–10]	8.6 ± 1.41 [0–10]	97.9 ± 4.06 [75–100] 100 (95–100)	9.1 ± 1.39 [0–10]	8.7 ± 1.47 [0–10]	97.2 ± 4.19 [80–100] 100 (95–100)	8.5 ± 1.61 [0–10]	8.5 ± 1.34 [2–10]
Upper abdomen									
Left and right diaphragm	90.0 ± 6.92 [75–100] 90 (85–95)	8.3 ± 1.73 [1–10]	8.1 ± 1.85 [0–10]	91.5 ± 6.58 [80–100] 90 (90–95)	8.6 ± 1.68 [1–10]	8.2 ± 1.94 [0–10]	88.3 ± 7.18 [75–100] 90 (85–90)	7.9 ± 1.71 [2–10]	7.9 ± 1.72 [1–10]
Spleen	95.0 ± 5.4 [85–100] 95 (90–100)	8.6 ± 1.47 [4–10]	8.4 ± 1.51 [0–10]	94.6 ± 5.58 [85–100] 95 (90–100)	8.9 ± 1.44 [5–10]	8.6 ± 1.68 [0–10]	95.4 ± 5.42 [85–100] 97.5 (90–100)	8.3 ± 1.44 [4–10]	8.2 ± 1.29 [3–10]
Liver parenchyma*	99.2 ± 2.36 [90–100] 100 (100–100)	9.2 ± 1.26 [0–10]	8.8 ± 1.29 [0–10]	99.2 ± 2.77 [90–100] 100 (100–100)	9.4 ± 1.23 [0–10]	8.9 ± 1.44 [0–10]	99.2 ± 1.95 [95–100] 100 (100–100)	8.9 ± 1.24 [5–10]	8.8 ± 1.10 [5–10]
Liver surface	97.0 ± 6.12 [75–100] 100 (95–100)	8.7 ± 1.54 [1–10]	8.5 ± 1.45 [2–10]	99.6 ± 1.39 [95–100] 100 (100–100)	9.2 ± 1.32 [3–10]	8.8 ± 1.44 [2–10]	94.2 ± 7.93 [75–100] 97.5 (90–100)	8.3 ± 1.63 [1–10]	8.3 ± 1.42 [2–10]
Hepatic hilum*	89.8 ± 8.35 [75–100] 90 (80–100)	7.9 ± 1.89 [0–10]	7.7 ± 1.85 [0–10]	89.6 ± 8.77 [80–100] 90 (80–100)	8.3 ± 1.96 [0–10]	7.9 ± 1.96 [0–10]	90 ± 8.26 [75–100] 90 (85–96)	7.5 ± 1.71 [3–10]	7.5 ± 1.69 [2–10]
Lesser omentum*	90.4 ± 8.53 [70–100] 90 (85–100)	8.1 ± 1.84 [2–10]	8.2 ± 1.67 [1–10]	91.9 ± 7.78 [80–100] 90 (85–100)	8.6 ± 1.45 [4–10]	8.4 ± 1.42 [3–10]	88.8 ± 9.32 [70–100] 90 (80–95)	7.7 ± 2.08 [2–10]	7.9 ± 1.86 [1–10]
Upper abdomen, all six sites	93.6 ± 7.49 [70–100] 95 (90–100)	8.5 ± 1.69 [0–10]	8.3 ± 1.66 [0–10]	94.4 ± 7.02 [80–100] 100 (90–100)	8.8 ± 1.58 [0–10]	8.5 ± 1.69 [0–10]	92.6 ± 7.92 [70–100] 95 (90–100)	8.1 ± 1.72 [1–10]	8.1 ± 1.63 [1–10]
Lymph nodes									
Inguinal	98.2 ± 4.05 [85–100] 100 (100–100)	9.26 ± 1.34 [0–10]	8.9 ± 1.47 [0–10]	98.8 ± 3 [90–100] 100 (100–100)	9.4 ± 1.33 [0–10]	8.9 ± 1.48 [0–10]	97.5 ± 5.00 [85–100] 100 (99–100)	9.0 ± 1.35 [1–10]	9.0 ± 1.46 [1–10]
Abdominal (para‐aortic)	98.0 ± 3.54 [85–100] 100 (95–100)	8.96 ± 1.34 [1–10]	8.4 ± 1.46 [1–10]	99.2 ± 1.88 [95–100] 100 (100–100)	9.4 ± 0.92 [5–10]	8.8 ± 1.25 [5–10]	96.7 ± 4.44 [85–100] 97.5 (95–100)	8.5 ± 1.56 [1–10]	8 ± 1.56 [1–10]
Pelvic (para‐iliac)	95.0 ± 4.79 [85–100] 95 (90–100)	9.13 ± 1.27 [2–10]	8.7 ± 1.55 [2–10]	95.0 ± 4.08 [90–100] 95 (90–100)	9.4 ± 0.98 [5–10]	8.9 ± 1.34 [3–10]	95.0 ± 5.64 [85–100] 97.5 (90–100)	8.8 ± 1.45 [2–10]	8.5 ± 1.73 [2–10]
Lymph nodes, all three sites	97.1 ± 4.36 [85–100] 100 (95–100)	9.1 ± 1.33 [0–10]	8.7 ± 1.51 [0–10]	97.7 ± 3.6 [90–100] 100 (95–100)	9.4 ± 1.09 [0–10]	8.9 ± 1.36 [0–10]	96.4 ± 5.02 [85–100] 100 (95–100)	8.8 ± 1.47 [1–10]	8.5 ± 1.63 [1–10]
All five non‐resectable sites*	94.8 ± 7.18 [70–100] 100 (90–100)	8.5 ± 1.71 [0–10]	8.4 ± 1.60 [0–10]	95.4 ± 6.86 [80–100] 100 (90–100)	8.9 ± 1.52 [0–10]	8.5 ± 1.59 [0–10]	94.2 ± 7.53 [70–100] 95 (90–100)	8.1 ± 1.82 [0–10]	8.2 ± 1.59 [1–10]

Data for videoclips classified correctly are given as mean ± SD, [range] and median (interquartile range). Results for diagnostic confidence level and image quality are given as mean ± SD [range] of the numeric rating scale (NRS) (with NRS ranging from 0 to 10, where 0 = very uncertain and 10 = absolutely certain for confidence level, and 0 = very unsatisfactory and 10 = perfect for image quality). Medians were calculated from all site results by all observers, with no compartment‐wise summarization before computation.

* Infiltration of small intestine surface or mesentery, liver parenchyma (central or multisegmental metastasis surrounded by normal parenchyma between liver visceral surface and lesion), hepatic hilum or lesser omentum indicates non‐resectable tumor.

**Figure 1:**
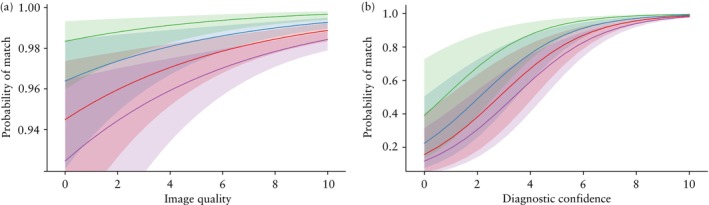
Marginal probability of correct classification of videoclips regarding ovarian cancer infiltration of four anatomical regions (pelvis (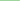), middle abdomen (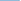), lymph nodes (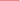) and upper abdomen (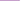)), depending on: (a) image quality as estimated by rater using a numeric rating scale ranging from 0 to 10, with level of diagnostic confidence set at 10 (i.e. median value for all 380 videoclips); (b) rater's diagnostic confidence expressed using a numeric rating scale ranging from 0 to 10, with image quality level set at 9 (i.e. median value for all 380 videoclips). Shading shows 95% confidence bands of the probability of correct classification. Results obtained using a generalized linear mixed model with random effects.

**Figure 2:**
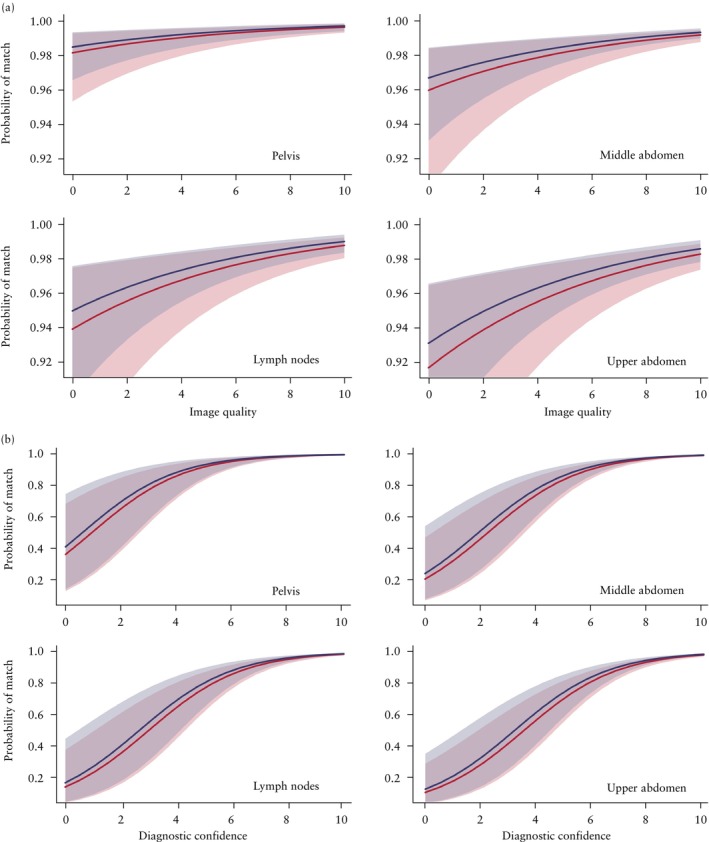
Marginal probability of correct classification of videoclips regarding ovarian cancer infiltration of four anatomical regions (pelvis, middle abdomen, lymph nodes and upper abdomen), depending on rater's level of experience (highly experienced (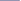) or less experienced (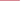)), as well as: (a) image quality as estimated by rater using a numeric rating scale ranging from 0 to 10, with level of diagnostic confidence set at 10 (i.e. median value for all 380 videoclips); and (b) rater's diagnostic confidence expressed using a numeric rating scale ranging from 0 to 10, with image quality level set at 9 (i.e. median value for all 380 videoclips). Shading shows 95% confidence bands of the probability of correct classification. Results obtained using a generalized linear mixed model with random effects. Highly experienced was defined as ≥ 10 years' experience in performing ultrasound examinations or ≥ 100 annual ultrasound examinations to estimate spread of ovarian cancer; less experienced was defined as < 10 years' experience in performing ultrasound examinations and < 100 annual ultrasound examinations to estimate spread of ovarian cancer.

According to the generalized linear mixed model with random effects, the probability of correct classification regarding ovarian cancer infiltration based on all 380 videoclips ranged from 0.956 to 0.975 (i.e. the expected percentage of correctly classified videoclips ranged from 95.6% to 97.5%), depending on the rater (Table 3). The model showed that the likelihood of correct classification of the 380 videoclips increased with increased image quality and diagnostic confidence, and was affected by anatomical region. The likelihood of correct classification was highest for sites in the pelvis, second highest for those in the middle abdomen, third highest for lymph nodes and lowest for sites in the upper abdomen (Table 4, Figures 1 and 2). In contrast, it was not affected by: the five sites that, if infiltrated, would indicate non‐resectable disease (*P* = 0.779); the level of expertise in gynecological scanning (EFSUMB Level II *vs* EFSUMB Level III) (*P* = 0.0652); the number of ultrasound examinations performed per year to assess spread of ovarian cancer (*P* = 0.533); the number of years performing ultrasound examinations to assess ovarian cancer spread (*P* = 0.679); the level of ultrasound experience in ovarian cancer staging (highly *vs* less experienced) (*P* = 0.413); the type of training in ultrasound assessment of the extent of ovarian cancer spread (self‐trained *vs* trained in a center specializing in ovarian cancer surgery) (*P* = 0.211); whether performing an ultrasound examination to evaluate ovarian cancer spread before surgery was part of their routine clinical practice (*P* = 0.373); or the type of ultrasound center in which the rater worked (oncological referral center *vs* other) (*P* = 0.826) (Table S7). The goodness‐of‐fit test for the generalized linear mixed model was statistically significant (*P* < 0.001).

**Table 3:** Probability of correct classification of 380 ultrasound videoclips regarding ovarian cancer infiltration by 25 raters (results of generalized mixed linear model with random effects)

Highly experienced raters		Less experienced raters	
Rater	Probability of correct classification	Rater	Probability of correct classification
Rater 24	0.9750960	Rater 4	0.9743136
Rater 21	0.9749915	Rater 7	0.9722473
Rater 1	0.9749219	Rater 14	0.9722097
Rater 12	0.9742882	Rater 2	0.9681312
Rater 19	0.9730182	Rater 18	0.9638373
Rater 11	0.9671114	Rater 13	0.9626686
Rater 8	0.9665980	Rater 20	0.9622156
Rater 5	0.9654149	Rater 17	0.9617259
Rater 25	0.9649107	Rater 6	0.9592044
Rater 16	0.9628723	Rater 23	0.9585553
Rater 3	0.9628345	Rater 15	0.9584678
Rater 9	0.9609936	Rater 22	0.9556784
Rater 10	0.9558279		
Overall	0.9676061	Overall	0.9641046

From final generalized linear mixed model, Bayes' prediction of probability of correct classification was calculated for each examiner using the estimated fixed parameters and taking into account the random effects present in the model.

**Table 4:** Factors affecting likelihood of correct classification of ultrasound videoclips regarding ovarian cancer infiltration (generalized linear mixed model with random effects)

Variable	Odds ratio (95% CI)	*P*
Log odds intercept	0.0616 (0.0168–0.2258)	< 0.001
Image quality (0–10, NRS)	1.1883 (1.0879–1.2980)	< 0.001
Diagnostic confidence (0–10, NRS)	1.7632 (1.5271–2.0359)	< 0.001
Upper abdomen (*vs* lymph nodes)	0.709 (0.4957–1.0137)	0.059
Middle abdomen (*vs* lymph nodes)	1.59 (1.0335–2.3741)	0.023
Pelvis (*vs* lymph nodes)	3.50 (1.9323–6.3414)	< 0.001

Lymph‐node assessment used as reference. Intercept corresponds to log odds for probability of correct classification of a videoclip when a (hypothetical) observer and a (hypothetical) videoclip with baseline characteristics (i.e. image quality = 0, level of confidence = 0 and location = ‘lymph nodes’) are considered. NRS, numeric rating scale.

There were no clinically important differences between the 12 less experienced and the 13 highly experienced ultrasound examiners in the observed percentage of correct classifications of videoclips regarding ovarian cancer infiltration in the four anatomical regions (Tables S2–S6) or in the probability of correct classification based on the generalized linear mixed model with random effects (Table 3). This was confirmed when image quality and level of diagnostic confidence of the rater were taken into account (Figure 2). There was also no difference between the highly experienced and less experienced examiners regarding the correct classification of videoclips from the five anatomical sites that, if infiltrated, would indicate non‐resectability of the tumor (Table S6).

The inter‐rater agreement of all 25 raters regarding the classification of anatomical sites as being infiltrated by cancer or not ranged from substantial (Fleiss kappa, 0.68 (95% CI, 0.66–0.71)) to very good (Fleiss kappa, 0.99 (95% CI, 0.97–1.00)), depending on the site (Table 5). Very good agreement (Fleiss kappa, 0.81–1.00) was obtained for 16 of the 19 sites and substantial agreement (Fleiss kappa, 0.61–0.80) for three sites, all in the upper abdomen (diaphragm, hepatic hilum and lesser omentum). With one exception, inter‐rater agreement between experienced examiners was similar to that between less experienced examiners: the inter‐rater agreement regarding tumor spread on the liver surface was better between the experienced raters than it was between the less experienced ones (Fleiss kappa, 0.98 (95% CI, 0.93–1.00) *vs* 0.77 (95% CI, 0.72–0.83)).

**Table 5:** Inter‐rater agreement regarding ovarian cancer infiltration according to anatomical site, as reflected by Fleiss kappa^9^

	Fleiss kappa (95% CI)		
Anatomical site	All raters (*n* = 25)	Highly experienced raters (*n* = 13)	Less experienced raters (*n* = 12)
Pelvis			
Anterior compartment	0.94 (0.92–0.97)	0.95 (0.90–1.00)	0.93 (0.87–0.98)
Posterior compartment	0.99 (0.97–1.00)	1.00 (0.95–1.00)	0.98 (0.93–1.00)
Rectosigmoid wall	0.98 (0.95–1.00)	0.98 (0.93–1.00)	0.97 (0.91–1.00)
Mesorectum and sigmoid mesocolon	0.96 (0.94–0.99)	1.00 (0.95–1.00)	0.92 (0.87–0.97)
Middle abdomen			
Greater omentum	0.96 (0.94–0.99)	0.98 (0.93–1.00)	0.94 (0.88–0.99)
Anterior abdominal wall	0.95 (0.92–0.97)	0.94 (0.89–0.99)	0.95 (0.90–1.00)
Left and right paracolic gutters	0.86 (0.84–0.89)	0.88 (0.83–0.93)	0.84 (0.78–0.89)
Colon surface	0.90 (0.87–0.92)	0.88 (0.83–0.93)	0.92 (0.86–0.97)
Small intestine surface*	0.90 (0.87–0.92)	0.96 (0.91–1.00)	0.82 (0.77–0.88)
Mesentery of small intestine*	0.92 (0.90–0.95)	0.91 (0.86–0.96)	0.94 (0.88–0.99)
Upper abdomen			
Left and right diaphragm	0.71 (0.68–0.73)	0.73 (0.68–0.78)	0.69 (0.64–0.74)
Spleen	0.82 (0.79–0.84)	0.81 (0.76–0.86)	0.82 (0.77–0.88)
Liver parenchyma*	0.97 (0.94–0.99)	0.97 (0.92–1.00)	0.97 (0.91–1.00)
Liver surface	0.88 (0.86–0.89)	0.98 (0.93–1.00)	0.77 (0.72–0.83)
Hepatic hilum*	0.68 (0.66–0.71)	0.67 (0.62–0.72)	0.69 (0.63–0.74)
Lesser omentum*	0.70 (0.67–0.73)	0.74 (0.69–0.79)	0.65 (0.59–0.70)
Lymph nodes			
Inguinal	0.93 (0.91–0.96)	0.96 (0.91–1.00)	0.90 (0.85–0.96)
Abdominal (para‐aortic)	0.92 (0.90–0.95)	0.97 (0.92–1.00)	0.88 (0.83–0.94)
Pelvic (para‐iliac)	0.88 (0.85–0.90)	0.90 (0.85–0.95)	0.87 (0.81–0.92)
All five non‐resectable sites*	0.83 (0.82–0.84)	0.85 (0.83–0.87)	0.81 (0.79–0.84)
All 19 sites	0.89 (0.88–0.89)	0.91 (0.89–0.92)	0.87 (0.85–0.88)

* Infiltration of small intestine surface or mesentery, liver parenchyma (central or multisegmental metastasis surrounded by normal parenchyma between liver visceral surface and lesion), hepatic hilum or lesser omentum indicates non‐resectable tumor.

## DISCUSSION

Our results show that, after a minimum of 6 months of supervised training in a center specializing in ovarian cancer surgery, EFSUMB Level‐II and Level‐III gynecological ultrasound examiners can correctly classify predefined anatomical sites as being infiltrated or not by ovarian cancer based on video recordings obtained by an experienced ultrasound examiner. They also show that inter‐rater agreement in such classification is substantial.

Our study has limitations. Owing to the study design, the raters' ability to assess correctly the extent of ovarian cancer using ultrasound and the inter‐rater agreement are likely to have been overestimated. Fifty percent of the videoclips from each anatomical site showed cancer infiltration and the raters knew that for each site there would be 10 videoclips with cancer infiltration and 10 without. This does not reflect clinical reality and it is highly likely to have inflated the kappa values. Moreover, the results are based on retrospective analysis of videoclips obtained by an experienced ultrasound examiner, which means that the image quality of most videoclips was high. Our results provide no information on the ability of the raters themselves to obtain ultrasound images of sufficient quality to evaluate tumor infiltration. However, interpretation of images forms an important part of making a diagnosis and corresponds to radiologists' interpretation of CT or MR images. Therefore, we believe that our results provide clinically valuable information. All raters were EFSUMB Level‐II or Level‐III gynecological ultrasound examiners and all had substantial experience of using ultrasound to assess the extent of ovarian cancer spread. Our results can be generalized only to ultrasound examiners with the same level of experience. On the other hand, only experienced and specially trained examiners should perform this type of examination, which should only be performed in dedicated cancer centers.

The ability of the raters to identify correctly cancer infiltration depended on the anatomical region, regardless of the rater's experience of performing ultrasound to estimate ovarian cancer spread. Their ability decreased progressively from pelvis to middle abdomen to lymph nodes to upper abdomen. This aligns with current evidence regarding the diagnostic accuracy of ultrasound for the preoperative assessment of ovarian cancer spread. The best performance of ultrasound has been reported for cancer infiltration in the pelvis, followed by the middle abdomen (best for greater omentum) and upper abdomen (poorest for lesser omentum, followed by diaphragm and liver surface)^2^, ^3^, ^12^. This may be explained by gynecologists being more familiar with pelvic than abdominal anatomy and by better image quality being obtained with transvaginal than with transabdominal scanning. Image quality is often poor in the subdiaphragmatic sites, especially in the absence of ascites^13^, and when the patient is less compliant with various breathing maneuvers that would help to improve image quality^14^. We found that, the higher the quality of the videoclip and the greater the rater's diagnostic confidence, the better the agreement with true cancer status, both for the less experienced and for the highly experienced examiners.

In our study, the probability of correct classification of cancer infiltration was not affected substantially by the rater's level of gynecological ultrasound expertise (EFSUMB level) or by their experience in performing ultrasound examinations to estimate ovarian cancer spread. However, all raters had high expertise in gynecological ultrasound (EFSUMB Level II or III) and all had substantial experience of using ultrasound to estimate the spread of ovarian cancer, albeit the level of experience differed between them. In agreement with this, in a retrospective study using analysis of videoclips, Pálsdóttir *et al*.^15^ demonstrated a similar ability of experienced and less experienced ultrasound examiners (after the latter had attended a 1‐h teaching workshop) to correctly identify cervical cancer (Fleiss kappa, 0.46 *vs* 0.46) and its depth of stromal invasion (Fleiss kappa, 0.53 *vs* 0.45). However, the experienced examiners were better at excluding parametrial invasion^15^. Similarly, using retrospective evaluation of ultrasound videoclips from endometrial cancer patients, Eriksson *et al*.^16^ found no difference between experienced and less experienced ultrasound examiners (after a 1‐h teaching workshop) in their ability to correctly identify deep myometrial invasion (Fleiss kappa, 0.52 *vs* 0.48). However, experienced examiners identified cervical stromal invasion of the endometrial cancer more accurately^16^.

Inter‐rater agreement in interpreting ultrasound images is clinically important: are the reported results reliable? In our study, the inter‐rater agreement was very good or substantial for all anatomical sites and, with one exception (liver surface), it was similar between experienced examiners and between less experienced examiners (poorer agreement between less experienced examiners). It was lowest for sites in the upper abdomen and highest for sites in the pelvis. Others have reported on the inter‐rater agreement for ultrasound assessment of the spread of cervical cancer and endometrial cancer using retrospective analysis of video recordings. Pálsdóttir *et al*.^15^ found inter‐rater agreement between experienced examiners to be superior to that between less experienced ones for parametrial involvement in cervical cancer (Fleiss kappa, 0.57 *vs* 0.44)^15^. Eriksson *et al*.^16^ reported better inter‐rater agreement between experienced than between less experienced ultrasound examiners regarding cervical stromal invasion of endometrial cancer (Fleiss kappa, 0.58 *vs* 0.45).

A common argument against the introduction of ultrasound into routine practice for estimating the extent of ovarian cancer spread and the prediction of non‐resectability of the tumor is that ultrasound is not a sufficiently good method for this purpose and that it is operator dependent. Our results support the idea that ultrasound examiners, after training, may be able to detect correctly and reliably infiltration of ovarian cancer in different anatomical sites, although their ability may have been overestimated in this study. There is a need for properly designed studies that evaluate the diagnostic accuracy and reliability of ultrasound for the estimation of ovarian cancer spread in clinical practice^17^, ^18^. In such studies, a consecutive sample of women suspected to have advanced ovarian cancer should be examined by two independent ultrasound examiners before surgery to assess the spread of the cancer. These examiners could have the same or different levels of experience of performing ultrasound examination for this assessment. Possibly, different patients could be examined by different pairs of examiners, so that the performance of more than two examiners and the inter‐rater agreement for more than one pair of examiners could be estimated. Such studies would assess the ability both to acquire and to interpret ultrasound images in a clinical setting. From a research point of view, it would be even better to have patients undergo examination by more than two ultrasound examiners, but it is unlikely that patients would accept this, and it would also be difficult to arrange this logistically.

In conclusion, we have shown that ultrasound examiners can classify correctly predefined anatomical sites as infiltrated or not infiltrated by ovarian cancer when studying video recordings obtained by an experienced ultrasound examiner, and that inter‐rater agreement in this classification is substantial. However, owing to the study design, our results regarding diagnostic accuracy and inter‐rater agreement are likely to be overoptimistic. We recommend that further studies be carried out to evaluate diagnostic accuracy and reliability in clinical practice.

## Supporting information


**Table S1** Ultrasound experience of raters
**Tables S2–S6** Observed percentage of videoclips classified correctly regarding cancer infiltration according to rater and site in the pelvis (Table S2), middle abdomen (Table S3), upper abdomen (Table S4), lymph nodes (Table S5) and overall (Table S6)
**Table S7** Covariates tested in the generalized linear mixed model with random effects

**Figure S1** Observed agreement with correct classification by 25 raters of 380 ultrasound videoclips regarding ovarian cancer infiltration in upper and middle abdomen, pelvis and lymph nodes. Pelvis included anterior and posterior compartments, rectosigmoid wall, mesorectum and sigmoid mesocolon; middle abdomen included greater omentum, anterior abdominal wall, left and right paracolic gutters, colon surface, small intestine surface and mesentery of small intestine; upper abdomen included left and right diaphragm, spleen, liver parenchyma, liver surface, hepatic hilum and lesser omentum; and lymph nodes included inguinal, abdominal (para‐aortic) and pelvic (para‐iliac) lymph nodes. Infiltration of small intestine surface or mesentery, liver parenchyma (central or multisegmental metastasis surrounded by normal parenchyma between liver visceral surface and lesion), hepatic hilum or lesser omentum indicates non‐resectability of tumor.

## Data Availability

Data available on request from the authors.
