# Supplementary material for: Ultrasound examiners' ability to describe ovarian cancer spread using preacquired ultrasound videoclips from a selected patient sample with high prevalence of cancer spread
Source: Ultrasound Obstet Gynecol. 2025 Apr 18;65(5):641–52. doi: 10.1002/uog.29208 (PMC12047678; doi:10.1002/uog.29208)
Supplement: Supplementary file 1 — Table S1 Ultrasound experience of raters Tables S2–S6 Observed percentage of videoclips classified correctly regarding cancer infiltration according to rater and site in the pelvis (Table S2), middle abdomen (Table S3), upper abdomen (Table S4), lymph nodes (Table S5) and overall (Table S6) Table S7 Covariates tested in the generalized linear mixed model with random effects [file UOG-65-641-s002.docx]

Table S1 **Ultrasound experience of raters**

| Rater with original study ID | Numbers of scans to assess ovarian cancer spread performed per year | Years performing ultrasound to assess ovarian cancer spread | EFSUMB level of ultrasound competence^8^ | Training in ultrasound assessment of ovarian cancer spread |
| --- | --- | --- | --- | --- |
| Highly experienced (n=13) |  |  |  |  |
| Observer 1 | <100 | 20 | Level III | Self-trained |
| Observer 3 | ≥100 | 6 | Level III | ≥ 6 months fellowship |
| Observer 5 | <100 | 15 | Level III | Self-trained |
| Observer 8 | ≥100 | 6 | Level III | ≥ 6 months fellowship |
| Observer 9 | ≥100 | 7 | Level III | ≥ 6 months fellowship |
| Observer 10 | ≥100 | 6 | Level III | ≥ 6 months fellowship |
| Observer 11 | ≥100 | 5 | Level III | ≥ 6 months fellowship |
| Observer 12 | ≥100 | 3 | Level II | ≥ 6 months fellowship |
| Observer 16 | <20 | 12 | Level III | Self-trained |
| Observer 19 | <20 | 15 | Level III | ≥ 6 months fellowship |
| Observer 21 | ≥100 | 5 | Level III | ≥ 6 months fellowship |
| Observer 24 | ≥100 | 7 | Level III | ≥ 6 months fellowship |
| Observer 25 | <50 | 10 | Level III | ≥6 months fellowship |
| Less experienced (n=12) |  |  |  |  |
| Observer 2 | <20 | 1 | Level II | ≥ 6 months fellowship |
| Observer 4 | <20 | 5 | Level III | ≥ 6 months fellowship |
| Observer 6 | <50 | 7 | Level III | Self-trained |
| Observer 7 | <100 | 3 | Level III | ≥ 6 months fellowship |
| Observer 13 | <20 | 1 | Level II | ≥ 6 months fellowship |
| Observer 14 | <20 | 8 | Level III | ≥ 6 months fellowship |
| Observer 15 | <50 | 2 | Level II | ≥ 6 months fellowship |
| Observer 17 | <10 | 1 | Level III | Self-trained |
| Observer 18 | <50 | 5 | Level III | ≥ 6 months fellowship |
| Observer 20 | <50 | 3 | Level III | ≥ 6 months fellowship |
| Observer 22 | <20 | 4 | Level II | ≥ 6 months fellowship |
| Observer 23 | <20 | 5 | Level II | ≥ 6 months fellowship |

*EFSUMB, the European Federation of Societies for Ultrasound in Medicine and Biology*^8^

*Raters were grouped as less or more experienced based on annual individual caseload and number of years performing ultrasound examinations to evaluate ovarian cancer spread (i.e., less experienced < 10 years performing ultrasound examinations to assess ovarian cancer spread and < 100 annual scans to assess ovarian cancer spread, highly experienced ≥ 10 years performing ultrasound examinations to assess ovarian cancer spread or ≥ 100 annual scans to assess ovarian cancer spread). According to EFSUMB*^8^*, Level III examiners are likely to spend most of their time undertaking gynecological ultrasound, or teaching, research and development in the field. Level II practitioners should have undertaken at least 2000 gynecological ultrasound examinations. The training required to attain this level of practice would usually be gained during a period of expert ultrasound training, which may be within, or after completion of, a specialist training program. To maintain competence at Level II, practitioners should perform at least 500 examinations each year. A Level I practitioner should have performed a minimum of 300 examinations under the supervision of a Level-II practitioner or an experienced Level-I practitioner with at least 2 years’ regular practical experience. To maintain Level I status, the practitioner should perform at least 300 examinations each year.*

| Table S2 Observed percentage of correctly classified videoclips regarding cancer infiltration by rater and site in the pelvis | | | | |
| --- | --- | --- | --- | --- |
| Observer | **Anterior compartment** | **Posterior compartment** | **Recto-sigmoid** | **Meso-sigma** |
| More experienced |  |  |  |  |
| Observer 1 | 20 (100%) | 20 (100%) | 20 (100%) | 20 (100%) |
| Observer 3 | 20 (100%) | 20 (100%) | 20 (100%) | 20 (100%) |
| Observer 5 | 20 (100%) | 20 (100%) | 20 (100%) | 20 (100%) |
| Observer 8 | 20 (100%) | 20 (100%) | 20 (100%) | 20 (100%) |
| Observer 9 | 20 (100%) | 20 (100%) | 20 (100%) | 20 (100%) |
| Observer 10 | 20 (100%) | 20 (100%) | 20 (100%) | 20 (100%) |
| Observer 11 | 20 (100%) | 20 (100%) | 20 (100%) | 20 (100%) |
| Observer 12 | 20 (100%) | 20 (100%) | 20 (100%) | 20 (100%) |
| Observer 16 | 20 (100%) | 20 (100%) | 20 (100%) | 20 (100%) |
| Observer 19 | 20 (100%) | 20 (100%) | 20 (100%) | 20 (100%) |
| Observer 21 | 18 (90%) | 20 (100%) | 19 (95%) | 20 (100%) |
| Observer 24 | 20 (100%) | 20 (100%) | 20 (100%) | 20 (100%) |
| Observer 25 | 19 (95%) | 20 (100%) | 20 (100%) | 20 (100%) |
| Median  (min; max) | **100%**  **(90;100)** | **100%**  **(100;100)** | **100%**  **(95;100)** | **100%**  **(100; 100)** |
| Less experienced |  |  |  |  |
| Observer 2 | 20 (100%) | 20 (100%) | 20 (100%) | 20 (100%) |
| Observer 4 | 18 (90%) | 20 (100%) | 20 (100%) | 20 (100%) |
| Observer 6 | 20 (100%) | 20 (100%) | 20 (100%) | 20 (100%) |
| Observer 7 | 18 (90%) | 20 (100%) | 18 (90%) | 19 (95%) |
| Observer 13 | 20 (100%) | 20 (100%) | 20 (100%) | 19 (95%) |
| Observer 14 | 20 (100%) | 20 (100%) | 20 (100%) | 20 (100%) |
| Observer 15 | 20 (100%) | 20 (100%) | 20 (100%) | 20 (100%) |
| Observer 17 | 20 (100%) | 20 (100%) | 20 (100%) | 20 (100%) |
| Observer 18 | 20 (100%) | 19 (95%) | 20 (100%) | 20 (100%) |
| Observer 20 | 19 (95%) | 20 (100%) | 20 (100%) | 18 (90%) |
| Observer 22 | 20 (100%) | 20 (100%) | 20 (100%) | 20 (100%) |
| Observer 23 | 20 (100%) | 20 (100%) | 20 (100%) | 19 (95%) |
| Median  (min; max) | **100%**  **(90; 100)** | **100%**  **(95; 100)** | **100%**  **(90; 100)** | **100%**  **(90; 100)** |
| Total |  |  |  |  |
| Median | **100%** | **100%** | **100%** | **100%** |
| Min; max | **(90; 100)** | **(95; 100)** | **(90; 100)** | **(90; 100)** |

| Table S3 Observed percentage of correctly classified videoclips regarding cancer infiltration by rater and site in middle abdomen | | | | | | |
| --- | --- | --- | --- | --- | --- | --- |
| Observer | **Greater omentum** | **Abdominal wall** | **Paracolic gutters** | **Colon peritoneal surface** | **Small intestine surface** | **Mesentery of small intestine** |
| More experienced |  |  |  |  |  |  |
| Observer 1 | 20 (100%) | 20 (100%) | 20 (100%) | 20 (100%) | 20 (100%) | 20 (100%) |
| Observer 3 | 20 (100%) | 20 (100%) | 18 (90%) | 19 (95%) | 20 (100%) | 20 (100%) |
| Observer 5 | 20 (100%) | 19 (95%) | 20 (100%) | 19 (95%) | 19 (95%) | 20 (100%) |
| Observer 8 | 20 (100%) | 20 (100%) | 19 (95%) | 20 (100%) | 20 (100%) | 18 (90%) |
| Observer 9 | 20 (100%) | 20 (100%) | 19 (95%) | 20 (100%) | 20 (100%) | 20 (100%) |
| Observer 10 | 20 (100%) | 20 (100%) | 20 (100%) | 20 (100%) | 20 (100%) | 20 (100%) |
| Observer 11 | 19 (95%) | 20 (100%) | 18 (90%) | 15 (75%) | 19 (95%) | 18 (90%) |
| Observer 12 | 20 (100%) | 18 (90%) | 20 (100%) | 20 (100%) | 20 (100%) | 18 (90%) |
| Observer 16 | 20 (100%) | 19 (95%) | 20 (100%) | 20 (100%) | 19 (95%) | 20 (100%) |
| Observer 19 | 20 (100%) | 20 (100%) | 19 (95%) | 20 (100%) | 20 (100%) | 20 (100%) |
| Observer 21 | 20 (100%) | 20 (100%) | 19 (95%) | 20 (100%) | 20 (100%) | 20 (100%) |
| Observer 24 | 20 (100%) | 20 (100%) | 19 (95%) | 20 (100%) | 20 (100%) | 20 (100%) |
| Observer 25 | 20 (100%) | 20 (100%) | 20 (100%) | 19 (95%) | 20 (100%) | 19 (95%) |
| Median  (min; max) | **100%**  **(95; 100)** | **100%**  **(90; 100)** | **95%**  **(90; 100)** | **100%**  **(75; 100)** | **100%**  **(95; 100)** | **100%**  **(90;100)** |
| Less experienced |  |  |  |  |  |  |
| Observer 2 | 20 (100%) | 20 (100%) | 20 (100%) | 20 (100%) | 20 (100%) | 20 (100%) |
| Observer 4 | 17 (85%) | 20 (100%) | 18 (90%) | 19 (95%) | 18 (90%) | 19 (95%) |
| Observer 6 | 20 (100%) | 20 (100%) | 19 (95%) | 20 (100%) | 19 (95%) | 19 (95%) |
| Observer 7 | 20 (100%) | 20 (100%) | 19 (95%) | 20 (100%) | 20 (100%) | 20 (100%) |
| Observer 13 | 20 (100%) | 19 (95%) | 18 (90%) | 19 (95%) | 18 (90%) | 19 (95%) |
| Observer 14 | 20 (100%) | 20 (100%) | 19 (95%) | 20 (100%) | 20 (100%) | 20 (100%) |
| Observer 15 | 20 (100%) | 20 (100%) | 19 (95%) | 20 (100%) | 20 (100%) | 20 (100%) |
| Observer 17 | 20 (100%) | 20 (100%) | 19 (95%) | 18 (90%) | 20 (100%) | 20 (100%) |
| Observer 18 | 20 (100%) | 18 (90%) | 19 (95%) | 20 (100%) | 18 (90%) | 20 (100%) |
| Observer 20 | 20 (100%) | 20 (100%) | 19 (95%) | 19 (95%) | 16 (80%) | 19 (95%) |
| Observer 22 | 20 (100%) | 20 (100%) | 20 (100%) | 20 (100%) | 20 (100%) | 20 (100%) |
| Observer 23 | 19 (95%) | 20 (100%) | 19 (95%) | 20 (100%) | 19 (95%) | 20 (100%) |
| Median  (min; max) | **100%**  **(85; 100)** | **100%**  **(90; 100)** | **95%**  **(90; 100)** | **100%**  **(90; 100)** | **97.5%**  **(80; 100)** | **100%**  **(95; 100)** |
| Total |  |  |  |  |  |  |
| Median | **100%** | **100%** | **95%** | **100%** | **100%** | **100%** |
| Min; max | **(85; 100)** | **(90; 100)** | **(90; 100)** | **(75; 100)** | **(80; 100)** | **(90; 100)** |

| Table S4 Observed percentage of correctly classified videoclips regarding cancer infiltration by rater and site in the upper abdomen | | | | | | |
| --- | --- | --- | --- | --- | --- | --- |
|  | **Spleen** | **Liver parenchyma** | **Liver surface** | **Hepatic hilum** | **Lesser omentum** | **Diaphragm**  **left and right** |
| More experienced |  |  |  |  |  |  |
| Observer 1 | 20 (100%) | 20 (100%) | 20 (100%) | 20 (100%) | 18 (90%) | 20 (100%) |
| Observer 3 | 17 (85%) | 20 (100%) | 19 (95%) | 16 (80%) | 19 (95%) | 19 (95%) |
| Observer 5 | 17 (85%) | 20 (100%) | 20 (100%) | 19 (95%) | 20 (100%) | 18 (90%) |
| Observer 8 | 20 (100%) | 20 (100%) | 20 (100%) | 16 (80%) | 16 (80%) | 18 (90%) |
| Observer 9 | 19 (95%) | 20 (100%) | 20 (100%) | 16 (80%) | 20 (100%) | 18 (90%) |
| Observer 10 | 18 (90%) | 20 (100%) | 20 (100%) | 18 (90%) | 18 (90%) | 20 (100%) |
| Observer 11 | 20 (100%) | 18 (90%) | 20 (100%) | 16 (80%) | 16 (80%) | 18 (90%) |
| Observer 12 | 20 (100%) | 20 (100%) | 20 (100%) | 20 (100%) | 20 (100%) | 16 (80%) |
| Observer 16 | 19 (95%) | 20 (100%) | 20 (100%) | 16 (80%) | 17 (85%) | 19 (95%) |
| Observer 19 | 19 (95%) | 20 (100%) | 20 (100%) | 20 (100%) | 20 (100%) | 16 (80%) |
| Observer 21 | 18 (90%) | 20 (100%) | 20 (100%) | 20 (100%) | 18 (90%) | 20 (100%) |
| Observer 24 | 20 (100%) | 20 (100%) | 20 (100%) | 18 (90%) | 20 (100%) | 18 (90%) |
| Observer 25 | 19 (95%) | 20 (100%) | 20 (100%) | 18 (90%) | 17 (85%) | 18 (90%) |
| Median  (min; max) | **95%**  **(85; 100)** | **100%**  **(90; 100)** | **100%**  **(95; 100)** | **90**  **(80; 100)** | **90**  **(80; 100)** | **90%**  **(80; 100)** |
| Less experienced |  |  |  |  |  |  |
| Observer 2 | 18 (90%) | 20 (100%) | 20 (100%) | 17 (85%) | 19 (95%) | 18 (90%) |
| Observer 4 | 19 (95%) | 20 (100%) | 19 (95%) | 17 (85%) | 20 (100%) | 17 (85%) |
| Observer 6 | 19 (95%) | 19 (95%) | 18 (90%) | 18 (90%) | 18 (90%) | 16 (80%) |
| Observer 7 | 20 (100%) | 19 (95%) | 19 (95%) | 20 (100%) | 14 (70%) | 18 (90%) |
| Observer 13 | 18 (90%) | 20 (100%) | 15 (75%) | 15 (75%) | 16 (80%) | 15 (75%) |
| Observer 14 | 20 (100%) | 20 (100%) | 20 (100%) | 20 (100%) | 16 (80%) | 18 (90%) |
| Observer 15 | 20 (100%) | 20 (100%) | 20 (100%) | 16 (80%) | 20 (100%) | 20 (100%) |
| Observer 17 | 20 (100%) | 20 (100%) | 20 (100%) | 18 (90%) | 18 (90%) | 18 (90%) |
| Observer 18 | 20 (100%) | 20 (100%) | 18 (90%) | 20 (100%) | 18 (90%) | 18 (90%) |
| Observer 20 | 18 (90%) | 20 (100%) | 20 (100%) | 17 (85%) | 16 (80%) | 17 (85%) |
| Observer 22 | 20 (100%) | 20 (100%) | 20 (100%) | 19 (95%) | 19 (95%) | 20 (100%) |
| Observer 23 | 17 (85%) | 20 (100%) | 17 (85%) | 19 (95%) | 19 (95%) | 17 (85%) |
| Median  (Min; max) | **97.5%**  **(85; 100)** | **100%**  **(95; 100)** | **97.5%**  **(75; 100)** | **90%**  **(75; 100)** | **90%**  **(70; 100)** | **90%**  **(75; 100)** |
| Total |  |  |  |  |  |  |
| Median | **95%** | **100%** | **100%** | **90%** | **90%** | **90%** |
| Min; max | **(85; 100)** | **(90; 100)** | **(75; 100)** | **(75; 100)** | **(70; 100)** | **(75; 100)** |

| Table S5 Observed percentage of correctly classified videoclips regarding cancer infiltration by rater and site for lymph nodes | | | |
| --- | --- | --- | --- |
|  | **Inguinal lymph nodes** | **Para-aortic lymph nodes** | **Pelvic lymph nodes** |
| More experienced |  |  |  |
| Observer 1 | 20 (100%) | 20 (100%) | 20 (100%) |
| Observer 3 | 20 (100%) | 20 (100%) | 19 (95%) |
| Observer 5 | 19 (95%) | 20 (100%) | 19 (95%) |
| Observer 8 | 20 (100%) | 20 (100%) | 18 (90%) |
| Observer 9 | 20 (100%) | 20 (100%) | 18 (90%) |
| Observer 10 | 20 (100%) | 20 (100%) | 20 (100%) |
| Observer 11 | 20 (100%) | 19 (95%) | 19 (95%) |
| Observer 12 | 20 (100%) | 20 (100%) | 18 (90%) |
| Observer 16 | 20 (100%) | 20 (100%) | 19 (95%) |
| Observer 19 | 20 (100%) | 20 (100%) | 18 (90%) |
| Observer 21 | 18 (90%) | 20 (100%) | 20 (100%) |
| Observer 24 | 20 (100%) | 20 (100%) | 20 (100%) |
| Observer 25 | 20 (100%) | 19 (95%) | 19 (95%) |
| Median  (Min; max) | **100%**  **(90; 100)** | **100%**  **(95; 100)** | **95%**  **(90; 100)** |
| Less experienced |  |  |  |
| Observer 2 | 18 (90%) | 19 (95%) | 20 (100%) |
| Observer 4 | 20 (100%) | 19 (95%) | 18 (90%) |
| Observer 6 | 20 (100%) | 19 (95%) | 20 (100%) |
| Observer 7 | 20 (100%) | 20 (100%) | 20 (100%) |
| Observer 13 | 19 (95%) | 19 (95%) | 18 (90%) |
| Observer 14 | 20 (100%) | 20 (100%) | 20 (100%) |
| Observer 15 | 20 (100%) | 20 (100%) | 18 (90%) |
| Observer 17 | 20 (100%) | 20 (100%) | 18 (90%) |
| Observer 18 | 20 (100%) | 20 (100%) | 20 (100%) |
| Observer 20 | 17 (85%) | 19 (95%) | 17 (85%) |
| Observer 22 | 20 (100%) | 17 (85%) | 20 (100%) |
| Observer 23 | 20 (100%) | 20 (100%) | 19 (95%) |
| Median  (min;max) | **100%**  **(85; 100)** | **97.5%**  **(85; 100)** | **97.5%**  **(85; 100)** |
| Total |  |  |  |
| Median | **100%** | **100%** | **95%** |
| Min; max | **(85; 100)** | **(85; 100)** | **(85; 100)** |

| Table S6 Observed percentage of correctly classified videoclips regarding cancer infiltration by rater and anatomical region | | | | | |
| --- | --- | --- | --- | --- | --- |
|  | **Pelvis** | **Middle abdomen** | **Upper abdomen** | **Lymph nodes** | **Non-resectability** |
| More experienced |  |  |  |  |  |
| Observer 1 | 80 (100%) | 120 (100%) | 118 (98.3%) | 60 (100%) | 98 (98%) |
| Observer 3 | 80 (100%) | 117 (97.5%) | 110 (91.7%) | 59 (98.3%) | 95 (95%) |
| Observer 5 | 80 (100%) | 117 (97.5%) | 114 (95%) | 58 (96.7%) | 98 (98%) |
| Observer 8 | 80 (100%) | 117 (97.5%) | 110 (91.7%) | 58 (96.7%) | 90 (90%) |
| Observer 9 | 80 (100%) | 119 (99.2%) | 113 (94.2%) | 58 (96.7%) | 96 (96%) |
| Observer 10 | 80 (100%) | 120 (100%) | 114 (95%) | 60 (100%) | 96 (96%) |
| Observer 11 | 80 (100%) | 109 (90.8%) | 108 (90%) | 58 (96.7%) | 87 (87%) |
| Observer 12 | 80 (100%) | 116 (96.7%) | 116 (96.7%) | 58 (96.7%) | 98 (98%) |
| Observer 16 | 80 (100%) | 118 (98.3%) | 111 (92.5%) | 59 (98.3%) | 92 (92%) |
| Observer 19 | 80 (100%) | 119 (99.2%) | 115 (95.8%) | 58 (96.7%) | 100 (100%) |
| Observer 21 | 77 (96.2%) | 119 (99.2%) | 116 (96.7%) | 58 (96.7%) | 98 (98%) |
| Observer 24 | 80 (100%) | 119 (99.2%) | 116 (96.7%) | 60 (100%) | 98 (98%) |
| Observer 25 | 79 (98.8%) | 118 (98.3%) | 112 (93.3%) | 58 (96.7%) | 94 (94%) |
| Median  (min; max) | **100%**  **(96.2; 100)** | **98.3%**  **(90.8; 100)** | **95%**  **(90.0; 100)** | **96.7%**  **(96.7; 100)** | **96.0%**  **(87.0;100)** |
| Less experienced |  |  |  |  |  |
| Observer 2 | 80 (100%) | 120 (100%) | 112 (93.3%) | 57 (95%) | 96 (96%) |
| Observer 4 | 78 (97.5%) | 111 (92.5%) | 112 (93.3%) | 57 (95%) | 94 (94%) |
| Observer 6 | 80 (100%) | 117 (97.5%) | 108 (90%) | 59 (98.3%) | 93 (93%) |
| Observer 7 | 75 (93.8%) | 119 (99.2%) | 110 (91.7%) | 60 (100%) | 93 (93%) |
| Observer 13 | 79 (98.8%) | 113 (94.2%) | 99 (82.5%) | 56 (93.3%) | 88 (88%) |
| Observer 14 | 80 (100%) | 119 (99.2%) | 114 (95%) | 60 (100%) | 96 (96%) |
| Observer 15 | 80 (100%) | 119 (99.2%) | 116 (96.7%) | 58 (96.7%) | 96 (96%) |
| Observer 17 | 80 (100%) | 117 (97.5%) | 114 (95%) | 58 (96.7%) | 96 (96%) |
| Observer 18 | 79 (98.8%) | 115 (95.8%) | 114 (95%) | 60 (100%) | 96 (96%) |
| Observer 20 | 77 (96.2%) | 113 (94.2%) | 108 (90%) | 53 (88.3%) | 88 (88%) |
| Observer 22 | 80 (100%) | 120 (100%) | 118 (98.3%) | 57 (95%) | 98 (98%) |
| Observer 23 | 79 (98.8%) | 117 (97.5%) | 109 (90.8%) | 59 (98.3%) | 97 (97%) |
| Median  (min; max) | **99.4%**  **(93.8; 100)** | **97.5%**  **(92.5; 100)** | **93.3%**  **(82.5; 98.3)** | **96.7%**  **(88.3; 100)** | **96.0%**  **(88.0;98.0)** |
| Total |  |  |  |  |  |
| Median | **100%** | **98.3%** | **94.2%** | **96.7%** | **96.0%** |
| Min; max | **(93.8; 100)** | **(90.8; 100)** | **(82.5; 100)** | **(88.3; 100)** | **(87.0;100)** |
| The median of each region is computed from the results of all sites measured by all observers, with no compartment-wise summarization before calculation (as opposed to Table 2, for which the median of each region was computed from the scores of all observers, pooled together). | | | | | |

**Table S7 Co-variates tested in the generalized linear mixed model with random effects**

| *Covariates* | *p - Value* |
| --- | --- |
| Non-significant: | **p>0.05** |
| Rater’s training (self-trained *vs* fellowship)* | 0.211 |
| Level of expertise in gynecological scanning (EFSUMB II *vs* III)† | 0.0652 |
| Number of ultrasound examinations performed per year to assess spread of ovarian cancer | 0.533 |
| Number of years performing ultrasound examinations to assess ovarian cancer spread | 0.679 |
| Level of ultrasound experience in ovarian cancer staging (highly *vs* less experienced)‡ | 0.413 |
| Routine performance of abdomino-pelvic ultrasound to evaluate ovarian cancer prior to surgery | 0.373 |
| Type of ultrasound center (oncological referral center *vs* others) | 0.826 |
| Anatomical sites that, if infiltrated, would indicate non-resectability§ | 0.779 |
| Significant: | **P≤0.05** |
| Image quality according to the rater | < 0.001 |
| Diagnostic confidence of the rater | < 0.001 |
| Anatomical region (pelvis, middle abdomen, upper abdomen, lymph nodes) | < 0.001 |

The theoretical statistical test level was set to 0.05. The R statistical software by R Core Team (2022) was used.

***** Raterś training, i.e. type of training in ultrasound assessment of the extent of ovarian cancer (self-trained, or training by trainers in a center specialized in ovarian cancer surgery.

†Level of expertise in gynecologic scanning according to the EFSUMB^8^, Level III examiners are likely to spend most of their time undertaking gynecological ultrasound, or teaching, research and development in the field. Level-II practitioners should have undertaken at least 2000 gynecological ultrasound examinations. The training required to attain this level of practice would usually be gained during a period of expert ultrasound training, which may be within, or after completion of, a specialist training program. To maintain competence at Level II, practitioners should perform at least 500 examinations each year.

‡Raters were grouped as less or more experienced based on annual individual caseload and number of years performing ultrasound examinations to evaluate ovarian cancer spread (i.e., less experienced < 10 years performing ultrasound examinations to assess ovarian cancer spread and < 100 annual scans to assess ovarian cancer spread, highly experienced ≥ 10 years performing ultrasound examinations to assess ovarian cancer spread or ≥ 100 annual scans to assess ovarian cancer spread).

§Five sites indicate non-resectable disease if affected by cancer: (1) diffuse small intestine carcinomatosis; (2) diffuse deep infiltration of the root of the small bowel mesentery; (3) diffuse carcinomatosis of the lesser omentum; (4) diffuse hepatic hilar infiltration; and (5) liver parenchymal metastases[.](https://isuog365.sharepoint.com/sites/EDMS-Journal/Restricted/HEV-Work/EDITING/UOG-2024-0409.R2-NEW-Daniela-new%20wf-check%20figs%20tables%20and%20fig%20legends/HB-Tables-S1-S7.docx#_ENREF_7)
