# Supplementary figures and images for: Ultrasound examiners' ability to describe ovarian cancer spread using preacquired ultrasound videoclips from a selected patient sample with high prevalence of cancer spread
Source: Ultrasound Obstet Gynecol. 2025 Apr 18;65(5):641–52. doi: 10.1002/uog.29208 (PMC12047678; doi:10.1002/uog.29208)

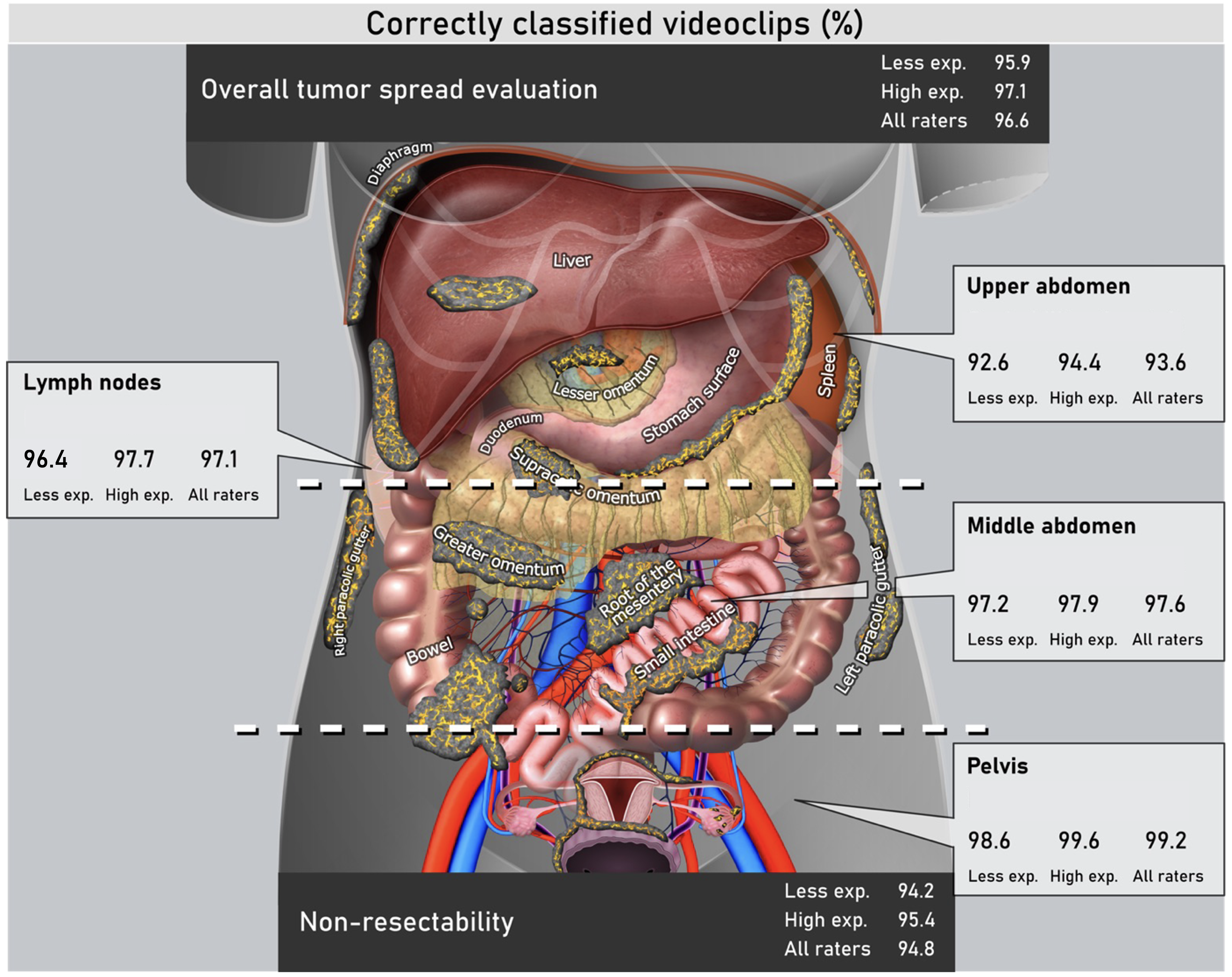

Supplement: Supplementary file 2 — Figure S1 Observed agreement with correct classification by 25 raters of 380 ultrasound videoclips regarding ovarian cancer infiltration in upper and middle abdomen, pelvis and lymph nodes. Pelvis included anterior and posterior compartments, rectosigmoid wall, mesorectum and sigmoid mesocolon; middle abdomen included greater omentum, anterior abdominal wall, left and right paracolic gutters, colon surface, small intestine surface and mesentery of small intestine; upper abdomen included left and right diaphragm, spleen, liver parenchyma, liver surface, hepatic hilum and lesser omentum; and lymph nodes included inguinal, abdominal (para‐aortic) and pelvic (para‐iliac) lymph nodes. Infiltration of small intestine surface or mesentery, liver parenchyma (central or multisegmental metastasis surrounded by normal parenchyma between liver visceral surface and lesion), hepatic hilum or lesser omentum indicates non‐resectability of tumor. [file UOG-65-641-s001.png]
